# Interleukin-4 (*IL4*) and Interleukin-4 receptor (*IL4RA*) polymorphisms in asthma: a case control study

**DOI:** 10.1186/1476-7961-3-15

**Published:** 2005-11-29

**Authors:** María Isidoro-García, Ignacio Dávila, Elena Laffond, Esther Moreno, Félix Lorente, Rogelio González-Sarmiento

**Affiliations:** 1 Molecular Medicine Unit, Department of Medicine, Faculty of Medicine, University of Salamanca, Campus Miguel de Unamuno, Salamanca 37008, Spain; 2 Department of Allergy, University Hospital of Salamanca, Paseo de San Vicente 58, Salamanca 37007, Spain

## Abstract

**Background:**

IL4/IL4RA pathway plays an important role in atopy and asthma. Different polymorphisms in *IL4 *and *IL4RA *genes have been described. Particularly, -33C>T*IL4 *and 576Q>R*IL4RA *SNPs have been independently associated to atopy and asthma. The purpose of this study was to analyse these polymorphisms in a population of patients with a well-characterized asthma phenotype.

**Methods:**

A total of 212 unrelated Caucasian individuals, 133 patients with asthma and 79 healthy subjects without symptoms or history of asthma or atopy and with negative skin prick tests were recruited. Lung function was measured by spirometry and asthma was specialist physician-diagnosed according to the ATS (American Thoracic Society) criteria and classified following the GINA (Global Initiative for Asthma) guidelines. Skin prick tests were performed according to EAACI recommendations. -33C>T*IL4 *was studied with TaqMan assay and 576Q>R*IL4RA *by PCR-RFLP technique. Hardy-Weinberg equilibrium was analysed in all groups. Dichotomous variables were analysed using χ^2^, Fisher exact test, Monte Carlo simulation test and odds ratio test. To model the effects of multiple covariates logistic regression was used.

**Results:**

No statistically significant differences between the group of patients with asthma and the controls were found when the allele and genotype distribution of -33C>T*IL4 *and 576Q>R*IL4RA *polymorphisms were compared. However, the T allele of the -33C>T*IL4 *SNP was more frequent in patients with persistent asthma. Multivariate analysis adjusted for age and sex confirmed that carriers of allele T had an increased risk of persistent asthma (OR:2.77, 95%CI:1.18–6.49; p = 0.019). Analysis of combination of polymorphisms showed that patients carrying both the T allele of -33C>T*IL4 *and the A allele of 576Q>R*IL4RA *had an increased risk of asthma. This association was particularly observed in persistent asthma [Fisher's p value = 0.0021, Monte Carlo p value (after 10^4 ^simulations) = 0.0016, OR:3.39; 95% CI:1.50–7.66].

**Conclusion:**

Our results show a trend of association between the genetic combination of the T allele of -33C>T*IL4 *and the A allele of 576Q>R*IL4RA *with asthma. This genetic variant was more frequently observed in patients with persistent asthma. As long as this study was performed in a small population, further studies in other populations are needed to confirm these results.

## Background

IL-4 is a Th2 cytokine that plays an essential role in IgE regulation. It triggers isotype switching from IgM to IgE, induces differentiation to Th2 phenotype on T cells and plays a critical role in the induction and maintenance of allergy.*IL4 *gene has been mapped to chromosome 5q31 where asthma and atopy have also been linked [1-3]. Evidence that *IL4 *polymorphisms are associated with total IgE levels and potentially with asthma and other allergy related phenotypes has been provided, although ethnical differences have been reported [4]. Specifically, the promoter region of *IL4 *has been associated with asthma phenotype [5] and a -33C>T polymorphism has been reported in this region [6]. An association between this polymorphism and asthma or atopy has been found, although this relation is still controversial [4,7-10]. IL-4 acts through the IL-4 receptor (IL-4R) that consists of two subunits, the α chain (IL-4Rα) and the γ chain (γc) [11,12]. IL-4Rα is a component of both the IL-4 and the IL-13 receptor complexes [13]. The *IL4RA *gene is located on chromosome 16p (16p12.1) [14], a region reported in linkage with atopy in different populations [15,16]. Several single-nucleotide polymorphisms (SNPs) have been identified in the coding region of the *IL4RA *gene, many of them resulting in aminoacid substitutions [17,18]. One of these polymorphisms, 576Q>R, consists of an A-to-G transition at nucleotide 1902, causing a change from glutamine to arginine at codon 576 (Q576R) in the cytoplasmic domain of the IL-4Rα. It has been reported that B-lymphocytes isolated from allergic patients bearing the 576Q>R mutation have an enhanced CD23 induction in response to IL-4 [19]. However, this result has not been confirmed by other authors [20]. Association of the 576Q>R polymorphism with the atopic phenotype has been described, but this relationship is still controversial [19,21-31]. Due to the central role of the IL-4/IL-4RA pathway in atopy and the scarce information about combinations of both genes in South European populations, we have analysed the -33C>T polymorphism of *IL4 *gene and the 576Q>R polymorphism of *IL4RA *gene in a Spanish population of patients with a well-characterized phenotype of asthma.

## Methods

### Subjects

We studied 212 unrelated Caucasian individuals, 133 patients and 79 controls, recruited from the outpatient Allergy Department of the University Hospital of Salamanca. The study was performed following the recommendations of the Ethical Committee of the University Hospital of Salamanca and informed written consent was obtained from each patient. Individuals who met all the following criteria were selected as controls: (i) no symptoms or history of asthma or other pulmonary diseases; (ii) no symptoms or history of atopy; (iii) negative skin prick tests to a battery of common aeroallergens (<1 mm wheal greater than saline) and (iv) absence of first-degree relatives with a history of asthma or atopy. Asthmatic patients were recruited if they had specialist physician-diagnosed asthma with the following characteristics: (i) at least two symptoms consistent with asthma (cough, wheeze and dyspnoea); (ii) either a positive bronchial hyperresponsiveness or a positive bronchodilator test defined as a ≥ 15% increase in baseline FEV1 after bronchodilator use; (iii) absence of other pulmonary disorders. Lung function was measured by spirometry according to ATS (American Thoracic Society) standards and severity of asthma was classified following GINA (Global Initiative for Asthma) guidelines. Asthma patients were grouped into intermittent and persistent by the clinical severity and into allergic and non allergic asthma by the clinical etiology.

Skin prick tests were performed according to EAACI recommendations with a battery of common aeroallergens that included *D pteronisynuss, D farinae, L destructor, T putrescentiae, A siro, G domesticus*, *E maynei*, mix of grasses, mix of trees, *P judaica, C album, A vulgaris, P lanceolata, O europaea, A alternata, C herbarum, P notatum, A fumigatus*, dog, cat, hamster, horse and rabbit dander and cockroach (ALK-Abelló, Madrid, Spain). Saline was used as negative control and histamine 10 mg/ml was used as positive control. Antihistamines were discontinued before skin testing according to published guidelines. Skin tests were considerer positive if at least one allergen elicited a wheal reaction of more than 3 mm of diameter after subtraction of the negative control. Patients were considered atopic if at least they had one positive skin test result. Total serum IgE was measured by a fluoroenzymeimmunoassay (Pharmacia Cap System^®^; Pharmacia, Uppsala, Sweden), according to the manufacturer's instructions.

### Genotyping analysis

After purification from peripheral blood leukocytes, DNA was amplified by polymerase chain reaction (PCR). Genotyping of -33C>T*IL4 *SNP was performed using a TaqMan assay in the ABI 7700 sequence detector and the allelic discrimination software Sequence Detector v1.7 according to the manufacturer's recommendations (Applied Biosystems). Primers and probes were obtained by means of the Assays-by-Demand SNP genotyping service of Applied Biosystems, Assay ID: C 16176215. Genotyping of the 576Q>R*IL4RA *polymorphism was performed according to a previously published assay [28]. Two oligonucleotides were used to amplify the polymorphic region of IL4RA: 5'-CCCCCACCACCAGTGGCTACC-3' and 5'-CCAGGAATGAGGTCTTGGAA-3' [24]. PCR reactions were carried out in a total volume of 25 μL, containing 50 ng of DNA and 12.5 μL of PCR Master Mix 2 × (Promega, Madison, Wisconsin). Amplification was performed with an initial denaturation step at 94°C for 5 min followed by 30 cycles of denaturation at 94°C for 1 min, primer annealing at 55°C for 1 min, and extension at 72° for 1 min. A final extension was carried out at 72° for 10 min. A blank amplification tube was always run to check for the presence of contamination. Strict rules were taken to avoid contamination. PCR reactions were prepared on a laminar flow hood and PCR products were examined in a different room. PCR products were digested for 4 h at 37°C with 1 U of MspI (New England Biolab, Boston, Massachusetts) restriction enzyme. After enzymatic digestion of the amplified fragments, the samples were analyzed by electrophoresis in 3% nusieve agarose gel. Control and patients were not genotyped in separated batched and the analysis was performed blindly with respect to case-control status.

### Statistical analysis

For case-control studies, the allele and genotype frequencies in patients with asthma were compared to a control non-asthmatic population. All the groups were tested for Hardy-Weinberg equilibrium using χ^2 ^analyses. The dichotomous variables were analysed using χ^2^, Fisher exact test, Monte Carlo simulation test (after 10^4 ^iterations) and odds ratio test. IgE levels were transformed to log_10 _values to produce a normal distribution for statistical analysis and analysed by ANOVA. To model the effects of multiple covariates on the dichotomous and continuous variables, logistic regression was used. In multivariate analysis, sex and age were included as potential covariates. A p-value less than 0.05 was considered statistically significant. Bonferroni correction was applied when appropriate. Case-control studies were also undertaken using combination of polymorphisms. Frequencies of combinations were estimated individually in controls and in samples to give the results of both single combinations and global data. For management of data, SHEsis software platform [32] and SPSS version 11 (SPSS Inc, Chicago, IL, USA) were used.

## Results

### -33C>T*IL4 *SNP

Characteristics of patients and controls are shown in Table 1. Genotype and allele frequencies are shown in Table 2. The -33T*IL4 *allele was found at a frequency of 0.15 in patients with asthma versus 0.09 in controls. No statistically significant differences between patients with asthma and controls were found. However, we observed an increase of the -33T IL4 allele in patients with persistent asthma compared to controls [Fisher's p value = 0.014, Monte Carlo p value (after 10^4 ^simulations) = 0.019]. Multivariate analysis of the genotypes adjusted for age and sex confirmed a trend of association of -33C>T*IL4 *polymorphism with an increased risk of persistent asthma (OR: 2.77, 95% CI: 1.18–6.49; p = 0.019). No differences were found in the group of subjects suffering allergic asthma compared to controls. Analysis of the total IgE levels failed to reveal any significant difference (p = 0.22), even when separate analysis for each gender was performed (data not shown).

**Table 1:** Demographic characteristics of patients

**Characteristic**	**Controls**	**Patients**	**P value**
**No. of subjects**	79	133	
**Age ± SD (y)**	40 ± 18	32 ± 17	0.001
**Sex (No.)**			
**Male**	28	56	0.38
**Female**	51	77	
**Log IgE ± SD**	1.36 ± 0.67	2.18 ± 0.71	<0.001

SD: standard deviation

y: years

**Table 2:** Genotype and allele frequencies of -33C>T*IL4 *and 576Q>R*IL4RA *SNPs

**Phenotype**			**Genotype**			**Allele**		**HWE p value**
	
**-33C>T IL4**	**N**	**Log IgE ± SD**	**CC**	**TC**	**TT**	**C**	**T**	
Controls	79	1.36 ± 0.67	0.81	0.19	0	0.91	0.09	0.35
Asthma	133	2.18 ± 0.71	0.70	0.29	0.01	0.85	0.15	0.15
Allergic Asthma	99	2.42 ± 0.56	0.69	0.30	0.01	0.84	0.16	0.24
Non-allergic Asthma	34	1.59 ± 0.76	0.74	0.26	0	0.87	0.13	0.37
Intermittent Asthma	54	2.39 ± 0.60	0.79	0.19	0.02	0.89	0.11	0.65
Persistent Asthma	79	2.09 ± 0.76	0.63	0.37*	0	0.82	0.18†	0.05
**576Q>R IL4RA**			**AA**	**AG**	**GG**	**A**	**G**	
Controls	79	1.36 ± 0.67	0.62	0.33	0.05	0.79	0.21	0.82
Asthma	133	2.18 ± 0.71	0.68	0.31	0.01	0.84	0.16	0.10
Allergic Asthma	99	2.42 ± 0.56	0.71	0.29	0	0.85	0.15	0.09
Non Allergic Asthma	34	1.59 ± 0.76	0.59	0.38	0.03	0.78	0.22	0.51
Intermittent asthma	54	2.39 ± 0.60	0.67	0.33	0	0.83	0.17	0.14
Persistent asthma	79	2.09 ± 0.76	0.68	0.30	0.01	0.84	0.16	0.35

HWE: Hardy-Weinberg Equilibrium

* Fisher's p value = 0.013, Monte Carlo p value (after 10^4 ^simulations) = 0.019

†Fisher's p value = 0.023, Monte Carlo p value (after 10^4 ^simulations) = 0.037

### 576Q>R*IL4RA *SNP

576R*IL4RA *arginine allele (G) was found at a frequency of 0.16 in patients with asthma versus 0.21 in healthy subjects (Table 2). We did not observe differences between patients and controls in allele or genotype frequencies. No association was detected with asthma phenotype or with asthma severity (Table 2). 576Q>R*IL4RA *polymorphism was not related to total serum IgE levels in our population (p = 0.35).

### Gene-gene interaction analysis

We did not detect differences in the global distribution of -33C>T*IL4 */ 576Q>R*IL4RA *combinations between the group of patients with asthma and the group of controls (Monte Carlo p value = 0.074) (Table 3). However, patients who were carriers of both the T allele of -33C>T*IL4 *and the A allele of *IL4RA *had an increased risk of asthma [Fisher's p value = 0.017, Monte Carlo p value (after 10^4 ^simulations = 0.012, odds ratio, 2.58; 95 % CI, 1.18–5.66].

**Table 3:** Distribution of combinations of -33C>T*IL4 *and 576Q>R*IL4RA *SNPs

**Genetic Variants**	**CTR**	**Asthma**	**Atopy**	**AA**	**PA**	**Monte Carlo p value (after 10^4 ^simulations)**			
						
						**CTR vs Asthma**	**CTR vs Atopy**	**CTR vs AA**	**CTR vs PA†**
**CQ**	0.73	0.71	0.72	0.73	0.68	0.584	0.813	0.900	0.281
**CR**	0.18	0.14	0.12	0.11	0.14	0.334	0.184	0.092	0.425
**TQ**	0.05	0.12	0.13	0.13	0.16	0.013*	0.016**	0.017***	0.002‡
**TR**	0.04	0.03	0.03	0.03	0.02	0.395	0.392	0.786	0.492

CTR, Controls; AA, Allergic Asthma; PA, Persistent Asthma

* Odds ratio: 2.59 95% CI: 1.18–5.66

** Odds ratio: 2.65 95% CI: 1.19–5.87

*** Odds ratio: 2.62 95% CI: 1.17–5.90

†Global distribution of genetic variants: Fisher's p value = 0.018, Monte Carlo p value (after 10^4 ^simulations) = 0.017

‡ Odds ratio: 3.39 95% CI: 1.50–7.66

Slight differences in the global distribution of genetic variants were detected between the group of patients with atopy and the group of controls [Fisher's p value = 0.05, Monte Carlo p value (after 10^4 ^simulations) = 0.045]. Patients who were carriers of both the T allele of -33C>T*IL4 *and the A allele of *IL4RA *had an increased risk of atopy [Fisher's p value = 0.013, Monte Carlo p value (after 10^4 ^simulations = 0.016, odds ratio, 2.64; 95 % CI, 1.93–5.87] (Table 3).

Differences in genetic variants distribution were also observed in the group of patients with allergic asthma compared to controls, although these differences did not reach statistical signification globally considered (Fisher p value = 0.053, Monte Carlo p value = 0.053). Again the combination of T and A alleles showed a trend of association [Fisher's p value = 0.016, Monte Carlo p value = 0.017, odds ratio, 2.62; 95 % CI, 1.17–5.90] (Table 3).

When we compared patients with persistent asthma to controls, significant differences in the global combination distribution were observed (Fisher's p value = 0.018, Monte Carlo p value = 0.017). Patients who carried both the T allele of -33C>T*IL4 *and the A allele of *IL4RA *had an increased risk of persistent asthma [Fisher's p value = 0.0021, Monte Carlo p value = 0.0016, odds ratio, 3.39; 95 % CI, 1.50–7.66] (Table 3).

## Discussion

### -33C>T*IL4 *SNP

We studied -33C>T*IL4 *and 576Q>R*IL4RA *polymorphisms in a well-characterized Spanish population of patients with asthma and in a healthy control population. Controls were older than patients allowing a longer period for asthma diagnosis to be made. When we analysed the -33C>T polymorphism independently, we did not detect significant differences in allele or genotype frequencies between the group of patients and the group of controls. Nevertheless, we observed a higher incidence of the T allele of -33*IL4 *in the group of patients with asthma (Table 2).

An association between -33C>T*IL4 *polymorphism and asthma or atopy has been previously reported, although this association is controversial [4,7-9]. In previous studies, no statistically significant association between -33C>T*IL4 *polymorphism and atopic dermatitis, bronchial hyperresponsiveness, atopic rhinitis and skin prick test reactivity was found [7,9]. However, a significant trend for an association between serum IgE levels and this SNP has been detected in children with positive skin prick tests, independent of asthma status [7].

As shown in table 2, we detected that the allele -33T*IL4 *is more frequent in patients with allergic asthma, although, we did not detect an association between this polymorphism and IgE levels.

It has been reported that polymorphisms within the promoter region of IL4 gene seems to correlate with enhanced IL4 activity [5,33], secondary to modification of *IL4 *gene transcription [34]. In this sense, it has been hypothesized that the T allele may be associated with severity of asthma [23,34,35]. In our study, a trend of association of -33T*IL4 *allele with persistent asthma was observed.

### 576Q>R*IL4RA *SNP

Analysis of 576Q>R*IL4RA *polymorphism did not reveal any association with asthma phenotype. It has been reported a relationship of the 576Q>R*IL4RA *SNP with the atopic phenotype, however this relationship is still controversial [19,21-31,36-38]. In a previous study, we did not find any association of this polymorphism with atopy or IgE levels, except in a specific of group of patients with family history of atopy [28].

Kruse et al [21] associated the R allele with lower total IgE values, but Hersey et al [19]found an association with high levels of total IgE. We did not detect any association with IgE levels. Genetic association studies are often difficult to interpret due to poor reproducibility in different populations [39,40]. This may well result, among other reasons, from the fact that these studies are focused only on one SNP [18,26,27] and that penetrance of the alleles may be influence by other factors [19].

### Gene-gene interaction analysis

When we analysed both polymorphisms simultaneously, differences between the group of patients with asthma and controls were detected. Particularly, patients who were carriers of both the T allele of -33C>T*IL4 *and the A allele of 576Q>R*IL4RA *had an increased risk of asthma.

Significant differences were observed in the group of patients with allergic asthma compared to controls. In addition, patients who carried both the T allele of -33C>T*IL4 *and the A allele of 576Q>R*IL4RA *had an increased risk of persistent asthma, in our population.

It has been previously suggested that both SNPs may modify the susceptibility to atopy or atopic asthma, independently. The importance of analysis of genetic variants has been previously illustrated, because the functional significance of a given polymorphism may only be evident in a specific setting of additional SNPs in the same or different genes [40]. It has also been pointed out that genetic association studies need careful classification of phenotypes, application of quality control in the performance of laboratory procedures and very stringent significant levels to assure reproducibility [20,39]., although it also may indicate true heterogeneity in gene-disease associations. We describe for the first time a specific genetic combination of *IL4/IL4RA *polymorphisms that shows a trend of association to persistent asthma in a South European population. As long as this study was performed in a small population, further studies in other populations are needed to confirm these results.

## Conclusion

We show a trend of association between -33C>T*IL4 *and 576Q>R*IL4RA *polymorphisms and asthma phenotype in a Spanish population. Patients who carried both the T allele of -33C>T*IL4 *and the A allele of 576Q>R*IL4RA *showed an increased risk of allergic asthma. In the population included in our study this combination was observed more frequently in patients with persistent asthma.

## Competing interests

The author(s) declare that they have no competing interests.

## Authors' contributions

MIG participated in the design of the study, carried out the molecular genetic studies, performed the statistical analysis and drafted the manuscript.

IDG participated in the design of the study, coordinated the clinical aspects of the study, helped to perform the statistical aspects and to draft the manuscript.

EL participated in the clinical aspects of the study.

EM participated in the clinical aspects of the study.

FL participated in the design and coordination of the study.

RGS conceived the study, participated in its design and coordination and helped to draft the manuscript.

All authors have read and approved the final manuscript.
